# The Experience of Return to Work Among People with Persistent Symptoms Following a Concussion: An Interview Study

**DOI:** 10.1007/s10926-025-10305-3

**Published:** 2025-06-30

**Authors:** Karin Vierø, Lisbeth Lund Pedersen, Jan Hartvigsen, Jette Primdahl

**Affiliations:** 1 University of Southern Denmark, Odense, Denmark; 2 Department of Sports Science and Clinical Biomechanics Faculty of Health Science, University of Southern Denmark, Odense, Denmark; 3 Svendborg Municipality, Svendborg, Denmark; 4 Center for Muscle and Joint Health, Department of Sports Science and Clinical Biomechanics Faculty of Health Science, University of Southern Denmark, Odense, Denmark; 5 Chiropractic Knowledge Hub, Odense, Denmark; 6 Department of Regional Health Research, University of Southern Denmark, Odense, Denmark; 7 Hospital Sønderjylland, University Hospital of Southern Denmark, Aabenraa, Denmark; 8 The Danish Center for Expertise in Rheumatology, Danish Hospital for Rheumatic Diseases, University Hospital of Southern Denmark, Sønderborg, Denmark

**Keywords:** Mild traumatic brain injury, Persistent symptoms, Health care, Return to work, Rehabilitation

## Abstract

**Purpose:**

This study aimed to explore the experiences of Danish adults experiencing persistent symptoms following a concussion with a focus on their return to work (RTW) process.

**Methods:**

We employed a phenomenological-hermeneutic approach, using semi-structured interviews with seven persons with persistent symptoms following a concussion from two Danish municipalities. Data were analyzed using reflexive thematic analysis.

**Results:**

The analysis derived four main themes: (1) *The importance of understanding and consideration from others*. The participant’s quality of life and RTW outcomes were influenced by the level of understanding and consideration they received from others. (2) *Balancing needs and limitations*. The participants struggled to balance their desire to work with their limited ability, which resulted in feelings of pressure and guilt towards their families. (3) *Experiencing random support*. The participants experienced random support from stakeholders and lack of guidance from the healthcare system. (4) *A sense of loneliness,* due to their inability to tolerate being around others.

**Conclusion:**

Recognition in daily life seem to play a key role in the RTW process for individuals with persistent symptoms following a concussion. They struggle to balance their limitations to match their desire to both work and family life. The support they received was often random and they felt lonely. The findings reflect the participant’s desire for structured and recognition-based rehabilitation and support to enhance their daily lives and facilitate the RTW process.

## Introduction

Concussions, also known as mild traumatic brain injury (mTBI), account for 70–90% of all head injuries worldwide [1]. The global incidence rate is 600 per 100,000 inhabitants per year [1]. In Denmark, the yearly incidence rate for concussion is 308 per 100,000, which is likely an underestimation, as the registration only includes individuals diagnosed in emergency departments and hospitals [2]. Incidence rates and associated costs are expected to rise in the future due to an aging population and the increasing use of motorised vehicles [3].

Diagnostic criteria define a concussion as resulting from the transfer of mechanical energy to the brain caused by external forces. These forces may involve head struck with an object, a hard object or surface, brain undergoing an acceleration/deceleration movement without direct contact between the head and an object or surface, and/or forces generated from blast or explosion [4].

Although most individuals with concussion experience spontaneous remission, an estimated 29% suffer from persistent symptoms even after 12 months [5]. The WHO ICD-11 diagnostic criteria from 2024 include a broad classification of persistent symptoms, i.e., Mild Neurocognitive Disorder, when symptoms such as headache, dizziness, fatigue, irritability, sleep disturbances and cognitive impairments persist for at least three months following a concussion, significantly affecting daily activities, and cannot be explained by other medical, neurological or psychiatric conditions [6, 7].

Individuals with persistent symptoms following a concussion often struggle to maintain their work capacity [8], which emphasizes the need for interdisciplinary and coordinated rehabilitation [9]. In Denmark, the Ministry of Employment’s guidelines state that the 98 Danish municipalities are responsible for restoring the work capacity of individuals receiving sickness benefits as quickly as possible [10]. The municipalities have developed a wide range of offers to support people with persistent symptoms following a concussion, from providing brain injury coordinators to no formal support at all [11].

Experiencing a concussion can be conceptualized as a critical event that may lead to the loss of identity, self-worth and perception of competence [12]. In addition, individuals with persistent symptoms often experience a perceived lack of understanding from others, which has been found to be associated with a low return to work (RTW) expectancy [13].

According to the German sociologist and philosopher Axel Honneth, recognition refers to the intersubjective acknowledgment of a person’s needs, rights and contributions, and is fundamental to self-realization and social belonging. Honneth outlines three spheres of recognition—love, rights, and solidarity—each playing a vital role in identity formation and social integration [14]. A lack of recognition within these spheres may undermine individual’s self-confidence, self-respect, and self-esteem [14]. There is a lack of evidence on how individuals with persistent symptoms experience daily life during the RTW process, which involves various stakeholders such as healthcare, municipal support, employers and relatives.

Therefore, the aim of this study was to explore how adult Danes with persistent symptoms following a concussion experience their daily lives with a focus on their RTW process.

## Methods

### Design

A qualitative study employing semi-structured interviews based on a phenomenological-hermeneutic approach was planned [15, 16]. The first author’s own previous experience with persistent symptoms following a concussion informed the initial assumption that the RTW process was influenced by support from home and collaboration with key stakeholders. She had personally experienced essential support from her spouse, as well as both the presence and absence of support from various stakeholders. The first author assumed that the participants might experience systemic neglect, emotional strain balancing personal goals with limited capacities, and that social support from their spouses, healthcare providers, employers, and municipalities would significantly shape the RTW process. During the interviews, she applied phenomenological reduction to bracket her preconceptions and remain open to the participant’s lived experiences.

### Participants

Individuals with persistent symptoms following a concussion were recruited from two Danish municipalities. Both municipalities offered programs in energy management. In Denmark, 27 out of the 98 municipalities currently offer energy management programs as part of vocational rehabilitation services [11]. While the content can vary between municipalities, these programs typically focus on education about concussion management and strategies for managing energy levels, such as graded return to work using pacing-strategies, task prioritization, and incorporating rest to reduce fatigue. Many municipalities also offer group sessions, providing participants with the opportunity to share experiences and frustrations. Such a service was offered in both municipalities represented in this study.

In one municipality, referrals to the energy management program were handled by the municipal job centre, whereas referrals were made by general practitioners (GPs) in the other one.

Danish-speaking individuals who met the diagnostic criteria for mTBI [4] and fulfilled the ICD-11 criteria for mild neurocognitive disorder [6] were eligible for inclusion. All the participants had received a clinical diagnosis of concussion from a healthcare professional, as shown in Table 3. Inclusion was based on self-reported persistent symptoms following a concussion lasting more than three months, significantly affecting daily life, and according to the gatekeepers, not attributable to other medical, neurological, or psychiatric conditions. In addition, participants had to be aged 18–64 years and been employed on a full-time basis prior to their concussion. They had to either currently be undergoing or recently have completed a RTW process.

The exclusion criteria encompassed being a student, being unemployed, working part time or having mental or physical challenges that affected the person’s work ability prior to the concussion.

In January and February 2024, the gatekeepers distributed invitations to a diverse group of individuals who met the eligibility criteria in both municipalities.

The invitations contained detailed information about the study along with the first author's contact details. Interested participants could then contact the first author for further information about the study and subsequently decide whether they wished to participate or not.

### Data collection

An interview guide was developed [17] in accordance with the aim of the study and based on evidence from the literature [5, 12, 13, 18–20]. It covered four themes and contained open-ended questions concerning the experiences of daily life with persistent symptoms and the RTW process (Table 1). The interview guide was pilot tested and subsequently refined in collaboration with a patient research partner (PRP).

**Table 1:** Interview guide

Theme 1: Experiences of daily life with persistent symptoms	Questions 1. How did you acquire your concussion? 2. Can you tell me a bit about your daily life after getting your concussion? 3. How do your symptoms affect your daily life? 4. Are there tasks you find difficult to manage now, due to your concussion compared to before? 5. How has your spouse/close relationships impacted your trajectory? 6. Do you think your close relationships influence how you cope with your daily life now?
Theme 2: Experiences related to health care system	Questions 7. What information and guidance have you received about your concussion? And about how to manage it? 8. Can you share a memorable episode from a doctor’s visit or your rehabilitation? 9. How did healthcare professionals ask about your daily life and what is important to you? 10. What worked well and not so well for you regarding the healthcare system? 11. What impact has it had on your daily life?
Theme 3: Influence of municipality	Questions 12. How does a typical meeting with your case worker unfold? 13. Can you describe how you experience your collaboration? 14. What worked well and not so well for you regarding collaboration with the municipality? 15. What does it mean for you in your daily life?
Theme 4: Influence of employer and the experience of the return to work (RTW) process	Questions 16. What work related process have you been through since getting your concussion? 17. Can you try to describe how your concussion has affected your ability to perform your job? 18. Are you currently attached to the labor market? If so, what do you do at your job? 19. Is there anything you think could improve your return to work? 20. What are your goals regarding your future work life? 21. How do you think your employer views your situation? 22. What does your employer and your work life mean to you in your daily life?
Follow-up and clarification of Themes 1–4	Question 23. Is there anything you would like to ask or share? Is this really #16? Please ask the authors

The pilot test also allowed the first author to refine the interviewing techniques and to determine whether the expected one-hour timeframe was realistic. Following each interview, information was gathered on characteristics such as months since injury, age, age of children living at home, co-habitant status and current labour market attachment.

The first author had not met the participants prior to the study. No assumptions regarding the participants experiences were expressed by the first author prior to or during the interviews.

Based on the participant’s preferences, the interviews were planned in the participant’s home, at a municipal location, or via telephone.

During the semi-structured interviews, an open minded approach using phenomenological reduction was used to achieve a pure description of the participant's experiences [21]. This type of interview can reflect the subject’s experience of reality outside of the interview context [17]. The interview style was relaxed, incorporating pauses to allow the participants time to reflect, recall and provide nuanced and rich descriptions [22].

Interviews were audio recorded and were transcribed verbatim by the first author using the software program, NVivo [14], to support a stringent and structured analysis. No fieldnotes were made during the interviews, but reflections were noted by the interviewer after each interview to capture the impression of the participant’s mood, body language, the context, and internal dynamics observed during the interview [17].

### Data analysis

The data were analyzed using reflexive thematic analysis (RTA) as described by Braun & Clarke, 2022 [23]. The RTA involved systematically deriving, analyzing and reporting patterns (themes) within the data [23]. In line with Braun and Clarke’s guidance, the thematic development was led by the first author, as reflexive thematic analysis does not require inter-coder reliability but emphasizes depth of engagement and reflexivity [23]. The co-authors contributed to the reflexive process by critically engaging with the themes to ensure that each was internally coherent and distinctive, capturing the nuanced complexities within the data and aligning with the overall aim of the study [23].

This study applied RTA from a contextualist epistemological position, which assumes that meaning is constructed through the interaction between researcher and participant and is always situated within specific social, cultural, and relational contexts [23].

To enhance the credibility of the analysis, a member checking process was conducted following the initial development of themes, where the preliminary themes were presented and discussed. Member checking, as described by Braun and Clarke [24], can be a valuable strategy for supporting credibility in qualitative research rooted in phenomenological traditions. This is especially relevant when employing a hermeneutic approach to explore subjective and situated experiences, as in contextualist RTA.

The six phases of RTA were employed in a non-linear iterative process involving movement both forwards and backwards between the parts and the whole throughout the analysis process [23, 25]. A detailed description of the applied phases of RTA is presented in Table 2. Appendix 1 provides an example of the analysis process. Appendix 2 provides a coding tree as a snapshot of the analysis.

**Table 2:** Overview of the applied Reflective thematic analysis

Phases in the RTA	Process
1: Familiarization	The first author transcribed all seven interviews. Subsequently, the first author listened to the recorded interviews again and reread the transcriptions. Familiarization notes were taken, based on (1) individual interviews and (2) the whole dataset
2: Generating initial codes	The first round of the inductive semantic coding process primarily captured the manifest meaning of the data. Consequently, 554 descriptive codes were developed, for example “a lonely experience” and “lack of consideration” The second round involved fine-tuning of the codes for precision and coherence. The codes were checked for consistency using NVivo and the number of codes was reduced to 168. New codes were composed of codes from the first round, offering a broader understanding and precision. For example, “The doctor lacked knowledge about concussion” was composed of “the doctor was not updated about concussions”, “did not receive much guidance from the doctor”, and “the doctor did not know much about it”
3: Generating candidate themes	An inductive approach created a flexible and contextually informed understanding of the data. The process was supported by the reflections noted by the interviewer after each interview. The process resulted in 12 candidate themes.
4: Generating initial themes	An overall mind map was developed from the 168 fine-tuned codes and was compared with the notes from the familiarization phase with the aim of selecting the most relevant descriptions and experiences. The initial theme development from phase three was extended through a process of re-engaging with all the coded data extracts and the entire dataset. A validity check of the quality and scope of the candidate themes was conducted, involving an iterative review of both the data and the preliminary analysis. This process aimed to enhance the richness and nuances of each theme and to ensure that each theme had its own distinct focus and boundaries. Various combinations of the 168 codes were tried until four main themes and five subthemes had been developed and described
5: Describing and naming themes	A separate mind map was created for each theme and each subtheme. All themes were refined and described and checked for sufficient nuance and for their contribution to the overall analysis and aim of this study
6: Producing the report	The themes were illustrated by selected quotes. The selected themes and citations were member-checked by the participants and the PRP. A patient research partner (PRP) confirmed the relevance of the candidate themes. Member-checking was conducted through telephone conversations with each of the seven participants to verify whether the derived themes reflected their experiences. All participants agreed with the derived themes and the selection of quotes, after which the themes were reported, compared and discussed in relation to existing literature and Axel Honneth’s theory of recognition. This theory encompasses three spheres: love (emotional recognition and personal relationships), rights (legal and social recognition as individuals with rights), and solidarity (social recognition within communities) [14]

*RTA* Reflective thematic analysis

Furthermore, the derived themes were discussed in relation to existing literature [5, 12, 13, 18, 26] and Axel Honneth’s theory of recognition [14].

### Ethical considerations

Potential participants were offered both verbal and written information about the study by the first author. Prior to their participation, all participants provided written informed consent. The PRP and the included participants were invited to take part in a member-checking phone call to verify if the derived themes accurately reflected their experiences. However, they were not invited to comment on the transcripts. The current study received permission to commence processing the pertinent personal data on February 9th, 2024, from the University of Southern Denmark Research & Innovation Office, (File Number 12.144). Data were managed and stored confidentially in accordance with the European General Data Protection Regulations and the Danish law for data protection [27]. In accordance with Danish law, a formal review by a scientific ethical board is not necessary for a qualitative study.

## Results

Seventeen individuals met the inclusion criteria across the two municipalities, and each was given an invitation by the respective gatekeepers. Of these, seven contacted the first author for further information about the study. All seven expressed their willingness to participate and provided written informed consent in February and March 2024, and were subsequently included in the study: four women and three men aged 34–61 years. The participants had experienced persistent symptoms from 12 to 60 months, were undergoing or had recently completed their RTW process, were on sick leave or had terminated work due to the persistent symptoms (Table 3). According to the gatekeepers, there were no notable differences between the participants and eligible individuals who had not expressed an interest to participate with respect to severity of the persistent symptoms, educational level or sex. According to the gatekeepers, the primary reason for not contacting the first author were forgetfulness, as well as lack of time and energy.

**Table 3:** Characteristics of the included participants

No	Sex	Age	Children living at home	Cause of trauma	Months since injury	Weeks from injury to concussion diagnosis	Who diagnosed the concussion	Months before knowledge of energy management	Occupation pre-injury	Employment status	Current working hours per week
1	Female	45–50	One teenager	Bicycle crash	54	1	Doctor at the hospital	50	Production worker	Sick leave	0
2	Female	35–40	Two pre-school children	Handball match (hit the back of the head on the floor)	12	1	GP	2,5	Medical secretary	Following a phased return plan at own workplace	30
3	Male	60–65	One teenager	Fireworks explosion	14	6	Neurologist	9	Social worker	Dismissed, on disability pension	0
4	Female	45–50	Three teenagers	Ball kicked to the back of the head	17	1	GP	8	School teacher	Following a phased return plan in another job function	18
5	Female	40–45	Two teenagers	Rear-end traffic collision	60	2	Doctor at hospital	3	Business advisor	Flexjob* at own workplace. Flexjob is a job with special conditions, considering the person's reduction in capacity to work, due to the disease. The person is only able to work less than half time (full time is 37 hours per week in Denmark). Age reported in range and age of children not specified to reduce the risk for identification of the informants.	15
6	Male	45–50	Two pre-school children	Motorcycle crash	19	46	Chief neurologist at Clinic	16	Pedagogue	Dismissed, currently going through a severance agreement	12
7	Male	35–40	Two pre-school children	Fall, hit forehead on pavement	26	1	GP	6	Marketing manager	Dismissed	0

*GP* General practitioner

The interviews were conducted in February and March 2024. All the participants opted for home-based face-to-face interviews. The interviews lasted 49–81 min (median 65).

## Findings

The approach used in the analysis was phenomenological-hermeneutic, which allows a nuanced interpretation of the initial inductive analysis [28].

**Fig. 1:**
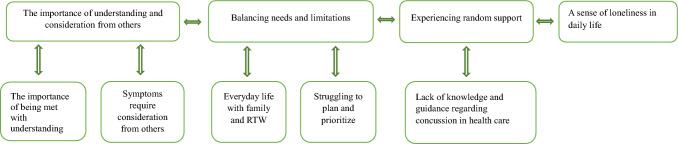
Themes and subthemes

The analysis derived four themes and five subthemes (Fig. 1).

In the following, the themes and subthemes are described and illustrated by selected quotes.

### Theme 1: The importance of understanding and consideration from others

The participants described that they were longing for people to understand their challenges and subsequently show the consideration they needed. They were anxious about whether their GP, social worker or employer would believe in their persistent symptoms and recognize their need for understanding and consideration in the RTW process.

*For my conscience it has been reassuring to encounter understanding and someone who doesn’t, uh, someone who believes that I genuinely want to return to work and that that’s what I’m striving for. Not to prolong the process and stay at home longer or, uh, that, it has really permeated the entire process.* (Participant 2)

### Subtheme 1a: The importance of being met with understanding

Encountering healthcare professionals, social workers and employers who demonstrated empathy led to a positive experience. Participants who met understanding in their surroundings regarding their persistent symptoms felt a sense of relief and gratitude, and experienced value in their daily lives.

*I could see some value in the offer that I could relate to, and it was just like wow, awesome, there really are people who want the best for me.* (Participant 7)

The participants described that an adapted RTW plan and an understanding of their challenges in everyday life meant everything to them in their RTW process.

*It* [an RTW plan] *cannot be described how much it has meant, and I’m also certain that it has contributed to making my process shorter, that I haven’t had to worry about that as well.* (Participant 2)

Lack of understanding resulted in a feeling of not being recognized. Participants who encountered distrust from their GP, social worker or employer described a sense of humiliation.

*Well, I’m a very stable worker, so the idea that I would, that I would use it as an escape route, well, I found that so humiliating.* (Participant 4)

Some participants described that their social worker concentrated on helping them in their RTW process but did not sufficiently address the challenges associated with their persistent symptoms in doing so.

The participants described how a lack of understanding also led to a feeling of pressure to perform beyond their capabilities, which was described as a scenario that contributed to extending the RTW process.

*I was constantly pressured into increasing the workload even though I didn’t feel ready...but also a sense of obligation that I have to do something because there are people who expect something from me.* (Participant 7)

Several participants described their everyday life as pure survival in an attempt to meet the employer’s agenda. It was an experience of feeling locked into the RTW plan, which led to negative thoughts and resulted in a sense of powerlessness and injustice.

*I just thought this was bloody unfair. And then I thought, argh, I should probably just have closed my eyes back then.* (Participant 1)

### Subtheme 1b: Symptoms require consideration from others

The persistent symptoms led to challenges in contributing at work on the same level that they had been able to prior to their concussion. The participants described how some stakeholders or family members expressed understanding, yet were unable to offer practical considerations due to contextual constraints. One participant described how her principal acknowledged her noise sensitivity but was unable to accommodate her need for consideration within the inherently noisy environment. Similarly, the participants with young children described how it was impossible to prevent noise at home, despite their need for quiet. In contrast, participants with older children or those working in quieter settings, experienced a level of understanding for silence, that more easily translated into practical considerations within their daily environments.

In addition to challenges with noise, the participants experienced difficulties with memory, concentration, word retrieval, reading and understanding written information, as well as with comprehending verbal communication.

Participants who experienced consideration from health care professionals, social workers, employers and colleagues described feeling included. Although their contributions did not match their previous work performance, some experienced recognition for their efforts.

*Just imagine, you could feel that you were a part of it, just by handing out fruit.* (Participant 5)

Other participants experienced that their challenges were not taken into consideration. Three participants had been hospitalized to get treatment for additional injuries sustained during the accident that caused their concussion. They described how exposure to loud voices, background noise, or sharing a room with a noisy patient during their initial hospital stay intensified their headache and led to increased irritation and frustration.

*One must consider that when you’ve hit your head, the sounds are just four times as loud in your head, I was furious, I simply had such a headache, couldn’t tolerate that the doctors and nurses in an intensive care unit would speak so loudly.* (Participant 1)

Later, in their everyday lives with persistent symptoms, the participants described finding it difficult to engage in medical consultations and meetings with caseworkers or employers because their concentration was impaired. The participants experienced mental blocks which led to poor retention and comprehension of information.

*You can ask me a perfectly normal question, and I can answer it. But then if you phrase it differently, somehow, I can’t understand it.* (Participant 3)

They described a wish for considerations such as shorter meetings, receiving fewer pieces of information at a time, and turning off background music.

### Theme 2: Balancing needs and limitations

The participants described that attachment to the labour market was highly valued. Conversely, they also described having difficulties sensing themselves, struggling to withdraw from social situations in due time, and grappling with their own boundaries. The participants described the experience of having difficulties balancing the desire to work and the lack of ability.

*I just need to get up and go to work and uh, well, cognitively I hadn’t even thought this is completely wrong, I mean, I haven’t been able to feel myself at all.* (Participant 6)

### Subtheme 2a: Everyday life with family and RTW

The aspiration for a daily routine encompassing full-time work and family life seemed to instigate a two-phased progression.

The first phase involved prioritizing work responsibilities as the participants were expecting the concussion to resolve swiftly.

*We actively prioritized that I should spend a significant amount of energy to return to work. I wanted to. It meant a lot to me.* (Participant 5)

Other participants described how they were unable to assert themselves or be attentive to their own needs. They experienced an imbalance between their own resources and the expectations regarding their gradual RTW plan.

*I used all my energy to be at work, so that resulted in me doing nothing but sleep when I got home, so I couldn’t contribute anything.* (Participant 6)

The experience of a pressured daily life was also linked to feelings of guilt towards their families. The participants experienced that their spouses ended up feeling overburdened with domestic chores, which devastated both the participants, their spouses and their interpersonal relationships.

*My partner is about to throw in the towel because she has to deal with everything herself. When I’ve been working, I’m just like feeling unwell, and that’s for the rest of the day.* (Participant 7)

The participants also described that their children were affected by the impact of their persistent symptoms. Their lack of energy after a day at work reduced their ability to engage with their children. Most of the participants said that they had sought psychological assistance to address the sense of guilt.

*I was playing with my daughter, and she was pressing her index fingers into my cheeks, saying, ‘Now you're happy again’, and I thought that I was feeling happy then, huh. That triggered something, and then I said to my boyfriend that I need to, and I need to have some tools. Dealing with, you know, when you fall into that dark place, uh... with the conscience, some tools to handle it better.* (Participant 2)

For some participants, it required a severance arrangement from their workplace to prompt a recognition of the extent to which they had pushed themselves and their loved ones due to their wish to work and their lack of work ability. This marked the inception of a second phase of their trajectory.

The participants described an altered balance in their daily lives, which facilitated a novel set of priorities. A desire arose for self-improvement and a reorientation towards prioritizing their families. However, a desire persisted to remain employed, albeit on reduced hours. The experience of guilt was gradually replaced by joy in everyday life in this second phase. The participants described that they contributed to their families and had the energy to care for their children and spouses. Moments of success, such as baking pancakes or going to the playground with the children, were highly valued.

*I actually took the kids to the playground myself. I had the energy for it, and since I can't remember the last time I had that, ehm, since the accident, you know, it’s really great that then, it was lovely.* (Participant 6)

### Subtheme 2b: Struggling to plan and prioritize

All participants described that their concussion affected their ability to plan even the most basic tasks. Any novelty, unpredictability or complexity was beyond their grasp. They were only able to undertake one simple task at a time, whether at home or at work. The participants described the condition as both demanding and frustrating. The participants struggled to cope with their challenges and found it difficult to manage the persistent symptoms.

*One thing. I can’t do something and then, well, then I have to finish it. Yes, and then the next thing. One thing at a time... I get so irritated with myself because of all the things I can’t do, that I used to be able to do. It also makes me hard on myself.* (Participant 3)

Several participants described courses in energy management as the municipality’s most beneficial offer, as it provided a new perspective on everyday life with persistent symptoms. They received specific guidance to manage their daily lives and learned how to prioritize their limited energy.

### Theme 3: Experiencing random support

The participants felt that it was random whether GP’s, social workers and employers showed understanding and offered support regarding their persistent symptoms. The participants felt that they did not receive the same support or recommendations as others in similar situations, which they found unsettling.

*A friend of mine, she has a colleague who is also a teacher, and she got a concussion and received completely different recommendations than I did. And I was just like ...that feels very unsafe.* (Participant 4)

It was random when and how the participants were diagnosed and random how they became aware of treatment options. The participants also experienced that it was random how they received information about the municipality’s offer regarding energy management.

*It was a coincidence that my boyfriend, at a music event, happened to speak with someone from the brain injury department. He happened to sit next to her and mentioned my situation, and she suggested I contact the person from the municipality’s brain injury department.* (Participant 2)

The experience of random support resulted in a feeling of uncertainty in their daily lives.

*Everything was random. EVERTHING I've figured out has been random. It’s terrible…I'm sure that’s also why my recovery process has been so long. (Participant 4)*

### Subtheme 3a: Lack of knowledge and guidance regarding concussion in health care

All participants described visiting GPs or seeking information at hospitals without receiving any guidance after sustaining their concussion.

*“No advice. I was just told, ‘You can go home now, your scan was fine,’ and when I mentioned I felt nauseous, they handed me a vomit bag and that was it.* (Participant 5)

The participants described their concerns regarding their persistent symptoms and the need for guidance. Seeking help but encountering ignorance was a frustrating experience.

*When all these things happen to your body, you get super scared because you’re constantly told,* ‘*There's nothing we can do to help you, there’s nothing we can do for you.*’ (Participant 7)

The participants expressed a wish for a coordinator who could guide them throughout the entire rehabilitation process, including assisting in planning their daily routines and managing challenges, supporting family members and facilitating the RTW process.

### Theme 4: A sense of loneliness in daily life

All participants experienced a sense of loneliness in their daily lives. They felt left alone, as if no one understood their challenges or were able to offer support. Also, the psychological burden of having to distance themselves from colleagues, family and friends due to their inability to handle prolonged social interactions was profound.

*For me, it’s very lonely, uh, also because I can’t cope with being around anyone, so it’s really difficult.* (Participant 3)

Energy management facilitated a prioritization of social engagement. Psychologically, the opportunity to interact with others served to alleviate feelings of loneliness temporarily.

*It’s lonely, and one can sink into such a frustrating, uh, spiral of feeling like it’s impossible to break free from again and whether it will ever get better, and yeah, then there are some days when I’ve prioritized spending time with my friends or that one time we went to a summerhouse, uh, for a weekend, and it just made such a difference mentally that I started prioritizing it.* (Participant 2)

The opportunity to connect with others facing similar challenges was perceived as supportive and as fostering an environment for exchanging experiences.

*Being together with others and hearing about their struggles and how they dealt with them gave a lot, and getting some ideas and realizing that you weren’t alone.* (Participant 5)

The participant’s experience of loneliness was influenced by the lack of support and understanding from people around them. Navigating the complexities of daily life felt overwhelming, particularly in the absence of external guidance. Hence, all the participants expressed a retrospective desire for early rehabilitation and support, yearning for support that would have facilitated a smoother adaptation to their new circumstances.

*There should have been someone to take me by the hand earlier and say, ‘We need to investigate this.’* (Participant 6)

## Discussion

The analysis derived four main themes and five subthemes representing the experiences of the RTW process and daily life among seven people with persistent symptoms following a concussion. Although each theme is presented separately, the analysis demonstrated that the themes are interconnected. It was evident that recognition is a central element across all themes and that all of the participants were affected by the impact of their symptoms on their families.

The participants who experienced a lack of understanding from stakeholders experienced a feeling of injustice, and they described how these issues prolonged their RTW process. Conversely, participants who experienced empathy and understanding from their GP, social workers, employers and colleagues reported improved RTW outcomes and quality of life.

These findings are in alignment with findings from Iverson et al. who found that among 102 Canadians who were slow to recover from concussions, a greater perceived injustice was associated with greater post-concussion symptoms, depression and negative expectations for recovery [13].

These findings are also in alignment with findings from Graff et al. who investigated the RTW process among 22 Danes with concussions 2–5 years post-injury. The study highlighted the importance of concussion-specific vocational rehabilitation and pointed out that GPs, social workers and employers are important stakeholders who may facilitate or obstruct the RTW process [8].

These findings emphasize the importance of emotional support for recovery and of reducing perceived injustice, which is related to the love sphere in Honneth’s theory of recognition [14]. The love sphere is crucial in fostering a supportive environment for those who are navigating complex recovery processes, such as the RTW process in people with persistent symptoms following a concussion.

The participants described that they struggled to recognize themselves after their concussion. They struggled to find a balance between their own desires to keep working and their actual capabilities on the one hand, and the expectations of either GP’s, social workers or employers regarding their RTW process on the other. This was a difficult and frustrating process for the participants, who expressed a need for psychological support to manage these circumstances.

These findings align with the findings by Sveen et al. who examined Michael Bury’s theory of biographical disruption in the context of the RTW process among 20 Norwegians with concussions [12]. Bury suggests that traumatic health events can disrupt an individual’s sense of identity, emphasizing the importance of diagnosis and professional support as essential components in separating oneself from the symptoms and beginning the process of identity reconstruction [29]. In agreement with Sveen et al.’s study, these findings highlight the participant’s experiences of having to handle identity changes and RTW challenges on their own [12].

The struggle with identity recognition and the need for comprehensive support resonate with Honneth’s theory of recognition and the importance of recognition across all three spheres of love, rights and solidarity as essential for a healthy identity formation [14]. Conversely, a lack of recognition in these three spheres may lead to symptoms similar to depression [14]. Recognizing an individual’s need for rehabilitation, particularly psychological support, is necessary to facilitate a successful balance of the RTW process and restoration of the sense of self.

The participants experienced loneliness due to insufficient support from GPs, social workers, and employers, as well as inability to engage in prolonged social interactions. Conversely, it was evident that participants who felt supported experienced a sense of recognition and inclusion. These findings align with the findings from a study by Snell et al. on patient perspectives on the recovery process by ten recovered and non-recovered New Zealanders with concussions. Snell et al. found that inadequate support from healthcare professionals and caregivers might lead to the experience of social isolation [26].

The experience of social isolation and loneliness resonates with Honneth’s spheres of love and solidarity, which underscores the crucial role of empathic support and understanding in alleviating social isolation and nurturing a sense of belonging and recognition [14].

The participants highlighted the benefits of the municipal energy management program, which taught them to prioritize their energy and activities in their daily life. However, they also described that the program was introduced randomly and often too late in their recovery process.

These findings align with those of Graff et al., who found that participants in their study often experienced excessive meetings with social workers that lacked a sufficient focus on concussion rehabilitation and that rehabilitation services were frequently offered too late [8].

The lack of recognition by social workers and municipal guidelines reflects a deficiency in Honneth’s sphere of rights, as the right to receive appropriate guidance, rehabilitation and recognition of challenges is overlooked. Similarly, the random and delayed access to energy management programs may indicate a lack of solidarity in terms of failing to align with participant’s needs and preferences [14].

The participants expressed frustration regarding the randomness of care and the lack of systematic guidance and rehabilitation. They described the omnipresent randomness as a source of uncertainty in their daily lives and emphasized the need for a coordinator from the onset of their concussion to provide personalized assistance, guidance in the RTW process and support for their families.

These findings align with the findings from Adams et al. who investigated eleven Americans with mild to severe traumatic brain injury and six primary caregivers in relation to coping and use of adaptive strategies [18]. The participants emphasized the need for professional support, information and structured planning and the caregivers suggested that healthcare professionals receive enhanced education to become able to assist patients with persistent symptoms and to support their families [18].

The absence of clear guidelines and the omnipresent randomness reflect deficiencies in all three spheres of Honneth’s recognition theory [14]. When any of these forms of recognition are not met, it results in imbalance and an incomplete realization of the good life [14].

The findings of this study may be further enriched by integrating additional relevant theoretical perspectives, providing a multidimensional understanding of the participant’s experiences.

Michael Bury’s concept of biographical disruption [29] highlighted how persistent symptoms following a concussion disrupted personal identity, with participants often reconstructing their identity alone or with minimal support from stakeholders [12]. This aligns with Arthur Frank’s narrative theory, which suggests that listening to others and sharing personal experiences facilitates trauma understanding, identity reconstruction, and the healing process [30]. The participant’s positive evaluations of the municipal energy management program echoed this theory, as they emphasized that being with others in similar situations, exchanging experiences, and realizing they were not alone significantly contributed to their sense of support and recovery.

Miranda Fricker’s theory of epistemic injustice [31] illuminates how struggles to be believed and understood can exacerbate marginalization, hinder recovery, and potentially in this case, prolong the RTW process. Such feelings of injustice may also affect an individual’s self-perception and sense of identity [31]. In line with Goffman’s theory of stigma, experiences of marginalization may lead to stigmatization, thereby increasing feelings of isolation and exclusion. [32]. According to the Danish philosopher, Knud Ejler Løgstrup, one is never in relation to another without holding something of their life in one’s hands, emphasizing the ethical responsibility to use professional power for the other’s benefit [33]. However, some of the participant’s experiences of epistemic injustice underscored how stakeholders failed to fulfill this responsibility, highlighting the importance of empathetic and supportive care.

Together, these theories emphasize the interconnectedness of identity disruption, perceived injustice, and storytelling in the RTW process. The biopsychosocial network model, recommended by the Danish Center for Concussion (2024) [7], complements this understanding by prioritizing personalized care that integrates medical, psychological, and social support. Specifically, the National Clinical Guideline for Non-Pharmacological Treatment of Persistent Symptoms Following Concussion (NKR)[34, 35] emphasizes the importance of an interdisciplinary approach, in which multiple professionals collaborate and coordinate treatment to ensure a biopsychosocial approach to the patient's needs. This includes recommendations for early information and counseling, graded physical activity, psychological interventions, and other non-pharmacological treatments [35], precisely as desired by the participants in this study.

Strengths and limitations

A limitation of the study was the relatively small number of participants. While a larger sample might have provided additional perspectives, the seven interviews provided rich and nuanced data and demonstrated thematic repetitions. This suggests that sufficient data were achieved to support meaningful analysis. Moreover, the findings are consistent with those of other international studies, further supporting their relevance. In line with Braun and Clarke’s definition of themes as capturing both complexity and richness within the dataset [23], this indicates that thematic saturation was achieved. However, it was not possible to explore the assumption regarding spousal support in the RTW process, as all the participants reported having supportive partners. It is therefore reasonable to assume that a larger sample would have provided greater variation regarding spousal support. Nonetheless, the data offered, supported the assumption that the RTW process is influenced by the level of cooperation with other stakeholders, which may help inform the development and evaluation of current practice. During the interviews, the first author’s professional knowledge and preconceptions may have influenced how the participants described their experiences, potentially limiting the depth of some narratives. One theme that lay outside these preconceptions was consideration. Although the first author had not anticipated the significance of this theme, the participants were nonetheless encouraged to elaborate. This serves as an example of how preconceptions were successfully bracketed in order to remain open to the participant’s lived experiences. Although preconceptions were deliberately bracketed, we acknowledge, in line with Braun and Clarke, that data cannot be generated in an epistemological vacuum [25]. Consequently, the analytical process was also informed by a hermeneutic approach, which positions the researcher’s interpretive role as central to the construction of meaning. Conducting repeated interviews, including sessions shortly after their concussion, might have provided more nuanced insights.

Another limitation was that all the participants were recruited from two municipalities that both offered energy management programs as part of their vocational rehabilitation services. This provided the opportunity to explore experiences with an existing intervention, assess its meaningfulness, and consider its potential for broader implementation in other municipalities. However, this may limit the transferability of our findings, particularly regarding individuals living in municipalities without such programs.

Some participants experienced memory and concentration difficulties. Despite this, they were able to describe their experiences. Questions such as whether recognition, identity, or experiences of guilt differ between male and female or whether male and female navigate the RTW process differently, are highly relevant. In our study, the participant’s experiences did not indicate sex-related differences. However, we recognize that such patterns might not derive without a targeted sampling approach specifically designed to explore sex and gender differences. Future studies employing purposeful sampling strategies focusing on gender dynamics could provide deeper insights into potential sex- and gender-specific experiences during the RTW process following a concussion.

A strength of the study was the diversity of participants, which enriched the findings. The first author’s commitment to phenomenological reduction ensured that the data collection process remained true to the participant’s authentic experiences. In addition, conducting interviews privately in the participant’s homes fostered a comfortable environment for them to share their personal experiences, minimizing distractions and the influence of family members. The involvement of a PRP facilitated the relevance of the patient perspective and enabled pilot testing of interview techniques, thereby enhancing the quality of the data collection process. Member checking by both the PRP and the participants validated the derived themes, ensuring that they accurately represented their experiences.

Future research could include more participants and incorporate triangulation to provide additional perspectives from key stakeholders like GPs, case workers and employers from multiple Danish municipalities.

## Conclusion

People with persistent symptoms following a concussion experienced challenges in balancing identity changes and personal needs with the demands of RTW plans and family life. Common experiences included lack of guidance, random support, and loneliness.

Those who received recognition from their stakeholders experienced a sense of value in daily life, leading to relief and gratitude, which they believed contributed to shortening their RTW process. In contrast, participants who did not experience recognition, reported feelings of humiliation, injustice, frustration, guilt towards their families and pressure to exceed their capabilities, which they believed contributed to prolong their RTW process.

Overall, our findings indicate a need for a more structured a biopsychosocial rehabilitative approach for individuals with persistent symptoms following a concussion. Not only to enhance the experience of RTW, but also to ensure support, recognition and the quality of life for themselves and their families.

## Figures and Tables

**Fig. 2:**
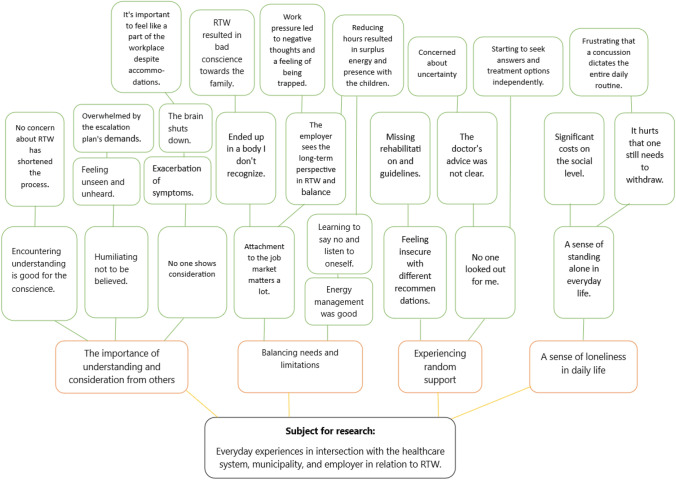
Coding tree. A visual presentation of the relationships between different codes and themes, helping to show the depth and complexity of the analysis

## Data Availability

No datasets were generated or analysed during the current study.

## Appendix 1

See Table

**Table 4:** example of the analysis process, deriving the theme “Experiencing random support”

Generating initial codes	Generating candidate themes	Theme	Quotes
A coincidence to find treatment The services provided by the brain injury coordinator were randomly discovered Seeking knowledge on my own Coming up with solutions myself The doctor should have informed me about treatment options Makes all the difference with a good leader The manager's focus on a quick return to work is too demanding Someone should have helped me	Coincidences in recovery Seeking help in vain Random support Coordinator wanted	Experiencing random support	“It was a coincidence that my boyfriend, at a music event, happened to speak with someone from the brain injury department. He happened to sit next to her and mentioned my situation, and she suggested I contact the person from the municipality’s brain injury department.” (Participant 2) “Everything was random. EVERTHING I've figured out has been random. It’s terrible…I'm sure that’s also why my recovery process has been so long.” (Participant 4) “So, it was purely a coincidence that I heard about her (K: Yes), and it's someone from the municipality I talked to. She deals with many people with concussions and tries to help me calm down because I'm constantly wondering what to expect” (Participant 7) “I have had to seek out help on my own. I don't feel like there is any clear guidance from my general practitioner on what they should recommend” (Participant 2) “But you're very much alone, you're on your own, you have to come up with solutions sometimes yourself” (Participant 3) “A friend of mine, she has a colleague who is also a teacher, and she got a concussion and received completely different recommendations than I did. And I was just like …that feels very unsafe.” (Participant 4) “There has also been immense support from my department. I hear that it’s a rare privilege, unfortunately, to be allowed to ease back into work over a year. Not all departments can accommodate that either, so it has certainly meant the world to me, for the process” (Participant 2)

4

## Appendix 2

See Fig. 2
